# Translating time-based research into team interventions: An actionable, evidence-based approach

**DOI:** 10.1017/cts.2021.874

**Published:** 2021-11-10

**Authors:** Susan Mohammed, David Schillinger

**Affiliations:** Department of Psychology, The Pennsylvania State University, University Park, Pennsylvania, USA

**Keywords:** Team science, temporal diversity, temporal leadership, team charters, team debriefs, team interventions

## Abstract

Temporal challenges are not only contextual in nature but manifest internally in teams when members enter the team with different temporal orientations (e.g., time urgency and pacing style). Researchers have demonstrated that temporal diversity has important implications for key team outcomes (performance, timeliness, and team conflict) across a range of samples and countries. Unfortunately, the practical implications of this research have yet to be unpacked. We respond to this need by developing an approach to translate temporal diversity research studies into actionable, evidence-based team interventions. Because journal articles are often deficient on actionable steps, whereas practitioner-friendly outlets tend to be deficient on scientific rigor, incorporating both criteria necessitates merging these literatures. Specifically, we delineate four main steps: (1) identify significant moderators, (2) match the moderators to scientifically based interventions, (3) design intervention tools with specific, actionable procedures, and (4) evaluate the effectiveness of the intervention tools by designing research studies. We believe the process we outline to marry actionable and evidence-based benchmarks is applicable to other research domains in team science beyond temporal research. It is our hope that this research will be a catalyst for further exploration of interventions that can help team members navigate temporal differences.

## Introduction

In a world where speed is a competitive asset, timeliness is a critical aspect of team performance. However, despite how important it is for temporal resources to be carefully managed, missed deadlines [1,2], scheduling, and time management [3] are routinely reported as significant problems in teams. Temporal challenges in teams include time pressure, fluctuating deadlines, and multifaceted coordination requirements [4]. Temporal demands are especially complex in translational teams in biomedical and health sciences who are tasked with fostering scientific breakthroughs that translate into real-world impact on health and wellbeing. Translational teams often must coordinate not only across members within teams, but also with other teams, departments, and organizations. Delays in translational teams not only hinder researchers but impede critical prevention and intervention efforts for our most vulnerable populations and at-risk communities.

Temporal challenges are not only contextual in nature but manifest internally in teams when members enter the team with different temporal orientations. For example, some individuals are relaxed toward time, whereas others are chronically hurried [5,6], reflecting differences in *time urgency*. On the *polychronicity* spectrum, some individuals prefer to focus on a single task at a time, whereas others prefer to work on several activities concurrently [7,8]. Diversity of *pacing styles* leads some people to complete work well before the deadline (early action) or work gradually over time (steady action), while others wait until the last minute to get started (deadline action) [9]. Temporal diversity is an umbrella term referring to intrateam heterogeneity on time-based individual differences such as time urgency, polychronicity, and pacing style [10].

What happens when members with diverse, temporal orientations must work interdependently in teams? As proposed theoretically, temporal diversity can result in conflicting or complementary influences on team performance [10]. On the negative side, time-based member differences may impede effective teamwork by creating ambiguity and dysfunctional conflict among members unless they are properly managed. For example, delays due to nontime-urgent members may aggravate time-urgent members, whereas strict schedules may be perceived as unnecessarily demanding by time-patient members. Polychronics may perceive monochronics as excessively rigid and inflexible when they decline to start a new task until an existing one is finished. Likewise, monochronics may view polychronics as scattered and fragmented when they dabble in several tasks simultaneously and fail to be on time [10]. To illustrate, one study found that the mix of time-urgent and time-patient as well as monochronic and polychronic members heightened disagreements over how temporal resources should be allocated [11]. Regarding pacing style, early and steady action members may perceive the “last minute heroics” of deadline action members to irresponsibly compromise performance quality by leaving too little room for reworks. Contrastingly, deadline action members may regard their style as efficient, because they can accommodate changes late in the task cycle [10].

On the positive side, however, temporal diversity can also improve team effectiveness by balancing contrasting team performance requirements [10]. Combining the speed of time-urgent members and the quality of time-patient members or the flexibility of polychronics and the focus of monochronics is theorized to produce superior performance when numerous criteria must be met [10]. Likewise, a mix of pacing styles may be well suited for coordinating complex tasks when action style members can begin, steady action style members can maintain project momentum, and deadline action style members can end [11]. To illustrate, one study found that temporal diversity resulted in both higher temporal conflict and increased performance [11].

What determines whether temporal diversity will produce harmful or helpful effects in teams? Although research is nascent, a growing number of studies have identified several contingency factors that moderate the relationship between temporal diversity and team outcomes, including the time-based activities of leaders [12] and whether team members are able to get on the same “temporal page” [13]. Researchers have demonstrated that temporal diversity has important implications for key team outcomes (e.g., performance [12,13], timeliness [13], and team conflict [14,15]) across a range of samples and countries (e.g., including information technology teams in India [12,13], student chef teams at a US culinary institute [11], and project teams in the Netherlands [16]). Time-based differences are theorized to be especially salient in settings involving time pressure and complex coordination [10]. Therefore, temporal diversity is also expected to have special relevance for translational science teams.

Given that coordination breakdowns or improvements, performance failures or successes, and costly delays or cost-saving efficiency can result from team members’ failure to converge temporally, how do we help team members navigate temporal diversity? Unfortunately, the practical implications of this research have yet to be unpacked. The emphasis of this emerging literature has been on demonstrating that temporal diversity matters for team processes and outcomes [12,13,15,16], so little is known about predictors and interventions. Although most of the journal articles cited above include a practical implications section, they are too brief and too vague. To provide clear guidelines for implementation. Thus, we are left with a need for concrete guidance on how to translate this emerging body of research into practical strategies for improvement.

**Fig. 1.**
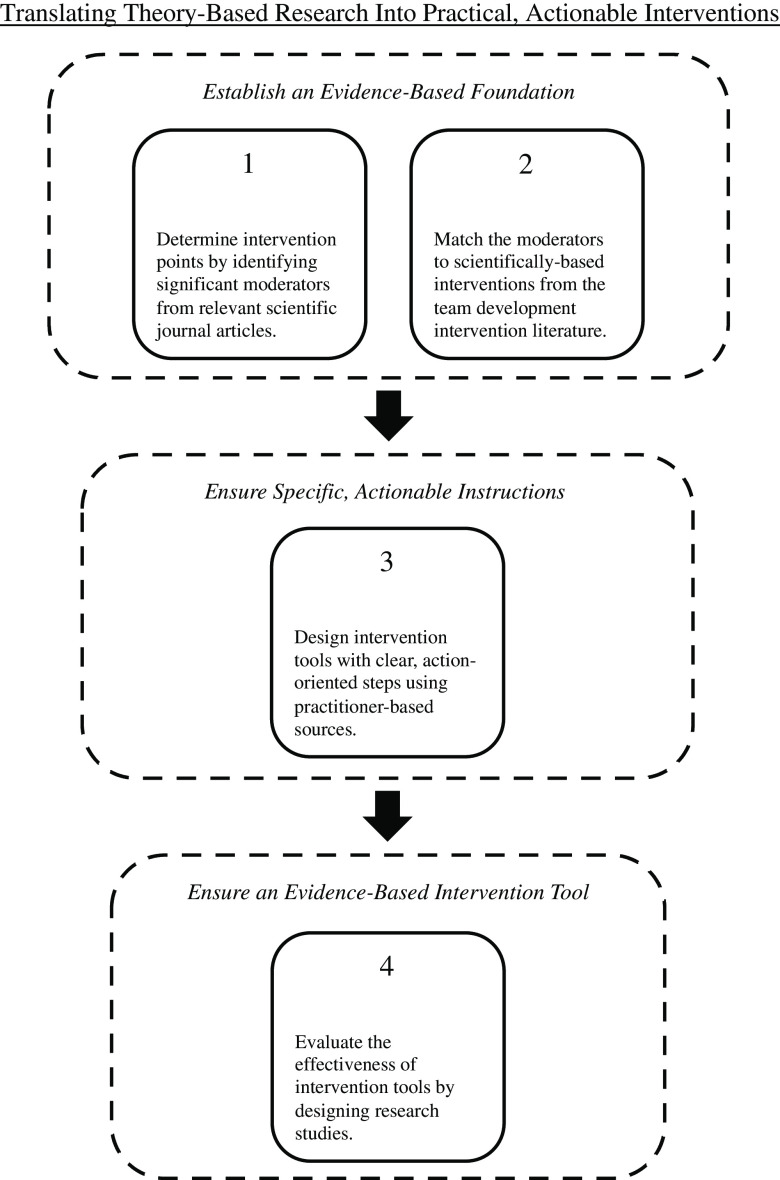
This figure outlines the four-step strategy for translating time-based theory and research into evidence-based, actionable interventions.

This article responds to this need by developing a procedure to translate temporal diversity research studies into actionable, evidence-based interventions. Because journal articles are often deficient on actionable steps, whereas practitioner-friendly outlets tend to be deficient on scientific rigor, incorporating both criteria necessitates merging these literatures. Specifically, we delineate four main steps. First, places are determined to intervene by identifying significant moderators from scientific journal articles. Second, the moderators are matched to scientifically based interventions from the team science intervention literature. Third, intervention tools are designed with specific, actionable procedures and instructions by drawing from practitioner-based sources. Fourth, the effectiveness of the intervention tools is evaluated by designing research studies to test for team process and performance improvements. As depicted in Fig. 1, the first two steps establish an evidence-based foundation, the third step ensures actionable procedures, and the last step ensures an evidence-based intervention tool. Below, we illustrate each step using temporal diversity research as our exemplar.

## Four Steps to Translate Research Studies into Actionable, Evidence-Based Interventions

### Step 1: Identify Significant Moderators

Moderators specify the conditions under which relationships hold, and so are an ideal starting point. Team science researchers would identify relevant moderators from scientific journal articles. In the case of temporal diversity, several moderators have been found that mitigate the negative effects of temporal diversity on team outcomes [12,13,16]. Specifically, we explore four moderators: temporal familiarity (knowledge of members’ time-based traits), action planning (delineating a course of action for intended work completion), temporal leadership (team leader behaviors that coordinate the pacing of task accomplishment), and shared temporal cognition (common understanding of the time-related aspects of executing collective tasks).

Gevers et al. [16] found that the relationship between pacing style diversity and team collaboration was positive only when both temporal familiarity and action planning were high. In addition, results from Mohammed and Nadkarni [12] showed that the effect of time urgency and pacing style diversity were more positive when team temporal leadership was high. Mohammed and Nadkarni [13] discovered shared temporal cognition to be a fourth moderator. Their study’s results revealed that shared temporal cognition weakened the negative effects of polychronicity diversity on team performance [13].

### Step 2: Match Moderators to Evidence-Based Team Interventions

Teams still fail frequently despite a voluminous team science literature that has identified myriad variables contributing to high-performing teams and the conditions under which they are more effective [17]. In response, research on team development interventions (TDIs) seeks to systematically translate scientific studies into tools, strategies, and actions that improve the performance trajectories of teams [18]. Meta-analytic evidence supports the effectiveness of several TDIs in improving team performance [19] and even reducing patient deaths in the healthcare context [20].

As team development consulting has popularized many resources that are not scientifically derived, a recent review of TDIs warned that it is critical to differentiate interventions that are evidence-based from those that have not been demonstrated to be effective [18]. Because evidence-based TDIs serve a critical role in increasing team effectiveness [15], we leverage them to guide our efforts toward translating temporal diversity research studies into actionable interventions.

Similar to Step 1, researchers familiar with team science research would identify TDIs from scientific journal articles. Team facilitators and practitioners would assess the relevance of moderators and TDIs based on their contextual knowledge of their team prior to implementation.

Specifically, the four moderators we identified from the temporal diversity literature (temporal familiarity, action planning, temporal leadership, and shared temporal cognition) are fittingly paired with three TDIs that have been shown to contribute to team improvements across a range of domains and settings [15,18]. As are described below, these three TDIs are team charters, leadership training, and team debriefs.

#### Temporal familiarity moderator matched to team charter TDI

Although temporal diversity may underlie performance problems in teams, members are often unaware of temporal differences in teams or their own time-based tendencies [10]. Because they often remain in the background of thought processes and behaviors, temporal characteristics are likely to be undetected or misattributed to more visible or common characteristics (e.g., lazy and uptight). Although subtle, empirical research has demonstrated that temporal diversity’s effects on team outcomes can be substantive [11–13,16]. Temporal diversity has therefore been described as “hidden but potent” [10]. Consequently, team members must first develop an awareness of their own, as well as members’, temporal orientations.

Fostering temporal familiarity can be accomplished via the TDI of a team charter, which is a written document developed and agreed upon by members to help jump-start a team by establishing ground rules for interaction and clarifying team direction [18]. Shown to increase team satisfaction, commitment, and performance [18,21], team charters should ideally be written when a new team is formed. While temporal familiarity is not traditionally included in team charters, it could easily be incorporated given that team charter best practices recommend asking members to identify their preferred work styles, strengths, and weaknesses [21]. Explicitly discussing temporal orientation in establishing team charters would accelerate a deeper understanding of members’ attitudes toward time and enable the development of coping strategies for handling temporal diversity.

Although helping team members understand their temporal orientation and that of their teammates is the first step, temporal interventions must go beyond an individual focus on temporal awareness. As recommended in the following paragraphs, incorporating team charters, leadership and/or team debriefs introduce key group communication and coordination mechanisms that have been shown to improve team performance in the team intervention literature (albeit without a temporal focus, which we now recommend).

#### Action planning moderator matched to team charter TDI

Action planning involves discussing goals, assigning roles, understanding team tasks, and considering constraints [16]. Because each of these parameters is commonly recommended for inclusion in a team charter [19], this TDI nicely operationalizes action planning. Team charters could readily be expanded to more explicitly incorporate conversations around member expectations for timing and scheduling, temporal constraints, and commitment to deadlines.

#### Temporal leadership moderator matched to leadership training TDI

Temporal leadership describes leader behaviors that aid in scheduling (e.g., deadline reminders), synchronizing workflow (e.g., coordinating teamwork to meet due dates), and allocating temporal resources (e.g., building in time for contingencies) [12]. Multiple studies show that higher temporal leadership contributes to higher team performance [12,22,23] and corporate entrepreneurship [24]. Given the demonstrated importance of temporal leadership, it is worthwhile to train leaders to improve their temporal leadership behaviors. Leadership training is a core TDI that has been meta-analytically found across 335 evaluation studies to not only contribute to a 25% improvement in learning, but a 28% improvement in on-the-job leadership behaviors, a 20% improvement in overall job performance, and a 25% improvement in organizational outcomes [15]. Building upon the evidence-based best practices for designing leadership training programs provides a solid foundation for incorporating temporality, such as synchronizing the team, building in contingency times, using temporal reminders, or creating schedules.

#### Shared temporal cognition moderator matched to team debriefs TDI

When teams have high levels of shared temporal cognition, members agree on specific deadlines, how quickly members should work to meet the deadline, and how work should be scheduled over time [25]. Research has demonstrated that shared temporal cognition exerts a strong, positive influence on team performance [13,26], coordination, and meeting deadlines [27]. While temporal leadership reflects a top-down strategy in which a team leader helps to coordinate members, so that work is accomplished on time, shared temporal cognition represents a bottom-up construct in which team members develop a joint temporal strategy [5]. As such, shared temporal cognition develops through communication about time, which may be triggered by critical events in the life of a team that then spark collective reflection [25]. For example, recurrent scheduling errors or missing team deadlines may prompt members to reactively diagnose what when wrong and proactively plan for how to prevent mistakes in the future. This practice aligns closely with the TDI referred to as team debriefs (also termed after-action reviews or reflexivity).

Team debriefs are structured learning experiences that encourage members to reflect on recent action that resulted in success or failure [28]. After discussing past action, uncovering problems, and celebrating successes, debriefs include steps to change future processes, thereby encouraging active self-learning and collaboration to derive specific ways to improve [15]. A meta-analysis of 46 samples concluded that debriefs increased team performance by 20%–25%, despite an average debrief time of only 18 min [28]. Building on the best practice recommendations from prior studies, debriefs could be expanded to include explicit reflection questions about how well the team coordinated actions to meet deadlines.

### Step 3: Design Interventions with Specific, Actionable Steps

Given that the scientific TDI literature emphasizes evaluation of intervention efficacy in its outcomes, it does not tend to specify how to conduct the interventions themselves. This results in a lack of clear guidelines for operationalizing the procedures.

Because the emphasis of the TDI literature is assessing the extent to which interventions positively predict team outcomes, practical guidelines are generally not written with the specificity needed for clear implementation. Rather, best practices outlined by this research typically include general advice such as “evaluate cognitive and/or skill-based content” for leadership training [15]. While it is important to incorporate these evidence-based recommendations, practitioner-based sources help to “fill in the blanks” regarding how to operationalize the suggestions. For example, “providing a sense of direction and developing plans to attain results” and “following up to ensure team commitments are met” [29] provide more actionable guidance.

**Fig. 2.**
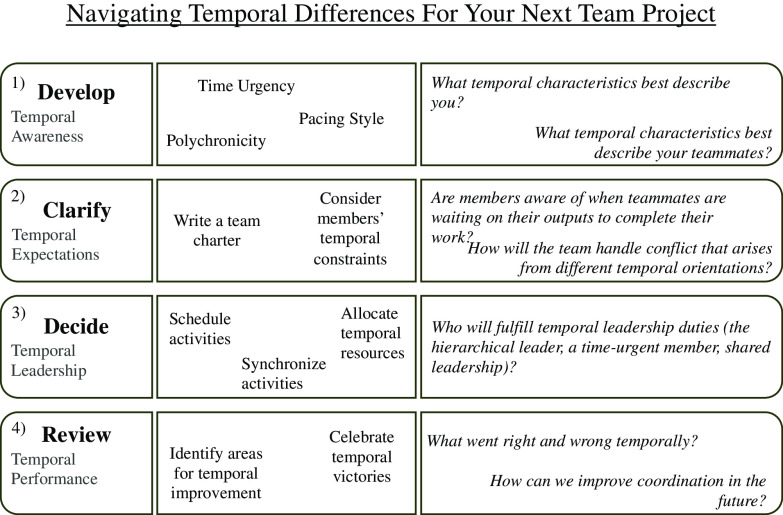
This figure outlines a four-step intervention for navigating temporal individual differences in teams and presents diagnostic questions for each step.

Suggestions for building on the above-mentioned TDIs with action steps are described below. Fig. 2 provides a sampling of some of the steps members can take and questions members can answer to successfully navigate temporal individual differences.

#### Temporal diversity awareness

Because temporal individual differences are not often part of the initial conversation or everyday language of getting work done in teams [30], it is not typical for team members to explicitly discuss their own or others’ orientations toward time. Members can develop an awareness of temporal differences in the team by completing measures of time urgency [6], polychronicity [8], and pacing style [9] and sharing results with each other (see Appendix for measures). It is also important to understand the strengths and weaknesses of diverse temporal orientations. For example, the benefits of monochronicity include concentration, full attention on one task at a time, adherence to plans, and punctuality [10]. However, because monochronic members interpret activities outside of the focal task as distractions to avoid, they may be slow in addressing teammate requests (e.g., emails and phone calls) until the focal task is finished [13]. Having a basic awareness of temporal diversity in the team is a prerequisite for members proactively developing strategies to handle tensions effectively when time-based differences emerge [10].

#### Temporal team charter

As part of completing a time-infused team charter, members can explicitly discuss what deadlines need to be met in order to achieve team goals, sequence tasks, celebrate making due dates, and establish consequences for missed deadlines. Through answering these questions, members can begin to form common expectations for timing and scheduling as well as a shared perspective of how conflicts arising from temporal diversity will be handled.

#### Training temporal leadership

Temporal leadership begins with an awareness that temporal diversity should be actively managed to minimize conflict and maximize performance [10]. With a basic understanding of team members’ temporal orientations, managers can begin to assign roles that match members’ time-based characteristics as much as possible. For example, assigning early pacing style members to start tasks, steady pacing style members to maintain project momentum over time, and deadline style members to complete tasks would be ideal [12]. Relying on time-urgent individuals to monitor speed and time-patient members to monitor quality may also be helpful [10].

Training should feature the temporal behaviors that have been found to improve performance, including reminding members of deadlines, prioritizing tasks, building in time for contingencies, and coordinating members to meet deadlines [12,22,23]. To illustrate, leaders need to consider members temporal constraints and how teammates can back each other up, so that deadlines are not compromised. In addition, leaders can foster an understanding of mutual dependencies among team members, so that members recognize when teammates are waiting on their outputs to complete their work. Detailing procedures for handing off performance responsibilities between members as projects progress can prevent unnecessary delays. Moreover, through monitoring temporal progress via check-ins, temporal leaders can be trained to recognize dysfunctional uses of time, assess whether the team is on track to meet deadlines, and adjust plans when necessary [15].

#### Temporal team debriefs

Because debriefs are more effective when they are structured [28], a series of time-based questions should be asked after a critical team event (e.g., major deliverable or completed milestone). For example, where did we meet and fail to meet our deadlines? What went right and wrong temporally? What caused our results (e.g., temporal leadership, handoff difficulties, role mismatches, and failure to communicate)? How can we improve coordination in the future? What are the important lessons learned regarding our time management?

#### Synergy across temporal intervention tools

Temporal team charters, temporal leadership training, and temporal debriefs nicely complement one another and can easily be integrated for maximum impact. For example, developing temporal awareness can help temporal leaders make more informed decisions and members to write a team charter that supports rather than undermines smooth collaboration within the team. A team charter can also formalize how members will determine who will fulfill temporal leadership duties (e.g., hierarchical leader, a time-urgent team member, and multiple team members) as well as when and how temporal team debriefs will be conducted.

### Step 4: Evaluate the Effectiveness of Interventions

While these proposed temporal interventions are grounded in science, they must be empirically tested to determine if they improve time-based outcomes, including coordination, timeliness, and performance. In addition, it would also be informative to examine which components of the intervention (temporal diversity awareness, temporal team charter, training temporal diversity, and temporal debriefs) are more and less effective and the optimal order by which they should be administered.

Team-based interventions evaluated by both academics *and* practitioners show greater learning and on-the-job behaviors compared to those evaluated by either academics *or* practitioners [15]. Supportive of the effectiveness of the scientist-practitioner model, academics, and industry experts each bring unique skillsets that produce superior results when combined. Therefore, the implications for the evaluation process are that university and industry stakeholders should work together to evaluate (as well as design and deliver) temporal interventions.

## Additional Considerations

Although our model begins with teams that have already been identified as needing intervention, we recognize that delineating the features of teams that might require a temporal intervention is important. Temporal diversity matters most in tasks that require high interdependence among team members [13]. Therefore, we recommend that temporal interventions be tested on teams that have to work closely with one another and in contexts where temporal demands are salient. In addition, because the effects of time-based diversity are theorized to depend on task characteristics [10], task demands and task complexity should be carefully evaluated.

Although our emphasis is on temporality, we acknowledge that other types of diversity (e.g., demographic and disciplinary) are clearly important and may interact with temporal differences in important ways. We also recognize that temporal diversity’s impact on team processes and outcomes will be influenced by contextual factors such as time pressure, temporal constraints, and the temporal climate of the department/unit/organization in which teams are embedded [31]. Furthermore, while we focus on time as an individual difference, we recognize that time is also culturally bound [32].

One potential barrier to implementing these tools is limited time, particularly for translational science teams facing challenging work. Although implementing the recommended temporal tools will take time, we expect that that their potential effectiveness will be worth the investment. Research has already demonstrated that temporal diversity has important implications for team performance, timeliness, and coordination [11,13,16]. Building on this work, we anticipate that temporal interventions can save teams the frustration and inefficiency of wasted effort, coordination breakdowns, dysfunctional conflict, and missed deadlines. However, future research to develop the interventions and to test their effectiveness is needed.

## Conclusion

This paper ventured into largely unexplored territory to translate emerging temporal diversity research into actionable and evidence-based interventions. Because temporal diversity has been shown to have important implications for key team outcomes such as team cooperation, timeliness, and performance, it is imperative to help teams manage time-based differences. For translational teams seeking to contribute to the team science literature, testing team interventions as outlined above would be a significant step forward. Thus far in the scientific literature, moderating influences have either reduced the negative effects *or* enhanced the positive effects of temporal diversity, with the latter less frequent than the former. Therefore, temporally based team interventions that both mitigate the harmful effects *and* harness the helpful effects are needed.

Given that translational teams aim to transport important and innovative treatment swiftly to populations in need, temporal diversity is especially important to address. Structuring teams to leverage diverse temporal orientations and avoid common pitfalls brought about by these differences could be beneficial for scientists and practitioners who manage the work demands and dynamic environments often faced by translational science teams.

Although we feature temporal research and practice in this paper, we believe the four-step process outlined in Fig. 1 is also relevant in other research domains in team science as a general recipe for creating evidence-based, actionable advice. However, further research is needed to test this conjecture. Our hope is that this research will be a catalyst to design, implement, and evaluate interventions to help members effectively navigate temporal individual differences in teams.

## Appendix: Scales to Measure Temporal Individual Differences

Time-Urgency [6]

The following self-report items are scored on a 1 to 5 Likert scale where 1 is *strongly disagree* and 5 is *strongly agree.* Items followed by (R) are reverse-scored.

*Task-Related Hurry*

1. I am unhurried at doing things. (R)
2. I like work that is unhurried and deliberate. (R)
3. I typically work unhurriedly and leisurely. (R)
4. People that know me well agree that I tend to do most things in a hurry.
5. I usually work fast.
6. I ordinarily work quickly and energetically.

*General Hurry*

1. I often feel very pressed for time.
2. I am usually pressed for time.
3. I am more restless and fidgety than most people.
4. I never feel in a rush, even under pressure. (R)
5. I find myself hurrying to get places even when there is plenty of time.
6. My family or close friends would rate me as definitely relaxed and easy going. (R)
7. Nowadays, I consider myself to be definitely relaxed and easy going. (R)
8. I am often in a hurry.

Polychronicity [8]

The following self-report items are scored on a 1 to 5 Likert scale where 1 is *strongly disagree* and 5 is *strongly agree.* Items followed by (R) are reverse-scored.

1. I prefer to work on several projects in a day, rather than completing one project and then switching to another.
2. I would like to work in a job where I was constantly shifting from one task to another, like a receptionist or an air traffic controller.
3. I lose interest in what I am doing if I have to focus on the same task for long periods of time, without thinking about or doing something else.
4. When doing a number of assignments, I like to switch back and forth between them rather than do one at a time.
5. I like to finish one task completely before focusing on anything else. (R)
6. It makes me uncomfortable when I am not able to finish one task completely before focusing on another task. (R)
7. I am much more engaged in what I am doing if I am able to switch between several different tasks.
8. I do not like having to shift my attention between multiple tasks. (R)
9. I would rather switch back and forth between several projects than concentrate my efforts on just one.
10. I would prefer to work in an environment where I can finish one task before starting the next. (R)
11. I don’t like when I have to stop in the middle of a task to work on something else. (R)
12. When I have a task to complete, I like to break it up by switching to other tasks intermittently.
13. I have a “one-track” mind. (R)
14. I prefer not to be interrupted when working on a task. (R)

Pacing Style [9]

The following self-report items are scored on a 1 to 5 Likert scale where 1 is *strongly disagree* and 5 is *strongly agree.*

*Deadline action pacing style*

1. I do not get much done on projects until the due date is close.
2. I generally do not work until there is time pressure from an approaching deadline.
3. I do most of the work on tasks in a short time before the deadline.

*Steady action pacing style*

1. I work steadily on tasks, spreading my work out evenly over time (e.g., 3 h per week until the deadline.)
2. I pace myself to work on projects a little bit every day or every week instead of doing several hours of work all at once.
3. I work in a slow, but steady manner to complete tasks.

*U-shaped action pacing style*

1. I put in more effort at the beginning of tasks as well as right before the deadline but am less active during the middle of the work cycle.
2. I invest most of my effort toward the beginning and end of projects.
3. The effort I put into projects is high at the start, low half-way through, and high again at the end.
